# Complete Killing of Agar Lawn Biofilms by Systematic Spacing of Antibiotic-Loaded Calcium Sulfate Beads

**DOI:** 10.3390/ma12244052

**Published:** 2019-12-05

**Authors:** Devendra H. Dusane, Jacob R. Brooks, Devin Sindeldecker, Casey W. Peters, Anthony Li, Nicholas R. Farrar, Scott M. Diamond, Cory S. Knecht, Roger D. Plaut, Craig Delury, Sean S. Aiken, Phillip A. Laycock, Anne Sullivan, Jeffrey F. Granger, Paul Stoodley

**Affiliations:** 1 Department of Microbial Infection and Immunity, The Ohio State University, Wexner Medical Center, Columbus, OH 43210, USA; devendra.dusane@osumc.edu (D.H.D.); brooks.922@buckeyemail.osu.edu (J.R.B.); sindeldecker.3@osu.edu (D.S.); peters.690@osu.edu (C.W.P.); li.5960@osu.edu (A.L.); Nicholas.Farrar@osumc.edu (N.R.F.); Scott.Diamond@beaumont.org (S.M.D.); knechtc2@ccf.org (C.S.K.); 2 Division of Bacterial, Parasitic, and Allergenic Products, Center for Biologics Evaluation and Research, Food and Drug Administration, Silver Spring, MD 20993, USA; Roger.Plaut@fda.hhs.gov; 3 Biocomposites Ltd., Keele Science Park, Keele, Staffordshire ST5 5NL, UK; cpd@biocomposites.com (C.D.); sa@biocomposites.com (S.S.A.); pl@biocomposites.com (P.A.L.); 4 Department of Orthopaedics, The Ohio State University, Wexner Medical Center, Columbus, OH 43210, USA; anne.sullivan@osumc.edu (A.S.); jgranger230@gmail.com (J.F.G.); 5 National Centre for Advanced Tribology at Southampton (nCATS) and National Biofilm Innovation Centre (NBIC), Department of Mechanical Engineering, University of Southampton, Southampton SO17 1BJ, UK

**Keywords:** antibiotic tolerance, biofilm, antibiotic-loaded bone cement, *pseudomonas*, *staphylococcus*, persister, periprosthetic joint infection

## Abstract

**Background:***Pseudomonas aeruginosa* (PA) and *Staphylococcus aureus* (SA) are the major causative agents of acute and chronic infections. Antibiotic-loaded calcium sulfate beads (ALCSB) are used in the management of musculoskeletal infections such as periprosthetic joint infections (PJI). **Methods:** To determine whether the number and spatial distribution of ALCSB are important factors to totally eradicate biofilms, ALCSBs containing vancomycin and tobramycin were placed on 24 h agar lawn biofilms as a single bead in the center, or as 16 beads placed as four clusters of four, a ring around the edge and as a group in the center or 19 beads evenly across the plate. Bioluminescence was used to assess spatial metabolic activity in real time. Replica plating was used to assess viability. **Results:** For both strains antibiotics released from the beads completely killed biofilm bacteria in a zone immediately adjacent to each bead. However, for PA extended incubation revealed the emergence of resistant colony phenotypes between the zone of eradication and the background lawn. The rate of biofilm clearing was greater when the beads were distributed evenly over the plate. **Conclusions:** Both number and distribution pattern of ALCSB are important to ensure adequate coverage of antibiotics required to eradicate biofilms.

## 1. Introduction

*Pseudomonas aeruginosa* (PA) is a Gram-negative, opportunistic bacterium associated with periprosthetic joint infections (PJI) [1]. *Staphylococcus aureus* (SA), a Gram-positive coccus, is a leading cause of skin [2,3], soft tissue [4,5], bloodstream [6], pneumonia [7], and bone and joint infections [8,9]. PA and SA have developed diverse strategies to respond and adapt to antibiotic stress, including the formation of antibiotic-tolerant biofilms [10,11].

Biofilms are microbial communities adhering to biotic or abiotic surfaces. In vitro studies and anecdotal clinical evidence suggests that bacteria within biofilms can resist killing at high antibiotic concentrations and often require a prolonged or repeated courses of antibiotics [12,13] at concentrations that are often outside the therapeutic window by systemic administration [14]. Bacteria within a biofilm become tolerant and can survive antibiotic treatments without necessarily having an acquired, heritable, resistance phenotype [15,16].

There are a number of mechanisms for biofilm antibiotic tolerance including restricted penetration of antibiotics, the physiological state of the cells (which can exhibit dormancy or slow growth due to nutrient limitation within the biofilm), and the formation of sub-populations of small colony phenotypes and persister cells (which are dormant even in areas of the biofilm where nutrients are available [17,18]). Repeated cycles of antibiotic exposure and resuscitation of persister cells on removal of antibiotics can lead to resistance [16]. Persistence of bacterial infection due to antibiotic tolerance is a huge problem in the treatment of chronic infections, with last resort antibiotics often becoming ineffective [19,20], leading to serious implications for patient outcome as well as economic loss [20,21].

Diverse mechanisms that confer resistance by PA and SA to antibiotics have been described [22,23]. The common example reported to confer antibiotic tolerance/resistance is inactivation of the antibiotic through the activity of enzymes produced by the bacterial cells [24,25]. The other mechanisms include decreased drug accumulation inside the bacterial cell via active efflux or diminished cell wall permeability [26,27,28,29]. Besides these strategies, growth within biofilms [10,11] or the emergence of persister cells [17,18] can contribute to survival in the presence of antibiotics. In addition, slow growing phenotypes are observed in both PA and SA. Reports suggests that small colony variants (SCVs) have been observed after antibiotic exposure, both in vitro and in vivo [30,31,32]. Hoffman et al. (2006) also showed the co-isolation of *P. aeruginosa* and *S. aureus* in infections such as cystic fibrosis. It has been reported that prolonged growth of *P. aeruginosa* or its exoproduct, 4-hydroxy-2-heptylquinoline-*N*-oxide (HQNO) with *S. aureus* would select for typical *S. aureus* SCV production. The *P. aeruginosa* exoproduct (HQNO) also protects *S. aureus* during coculture from being killed by tobramycin [31]. A recent study has shown the mechanism of antibiotic tolerance of biofilms during agar diffusion antimicrobial susceptibility testing was due to a switch from planktonic to biofilm mode of growth [33]. Generation of antibiotic tolerance and resistance has been associated with the failure of antibiotic treatment and relapse of bacterial infections; therefore, treatment strategies are necessary.

To treat these infections, in the case of patients with PJI, prevention strategies following total joint arthroplasty includes the use of antibiotic-loaded poly(methyl methacrylate) [34,35] or calcium sulfate (CaSO_4_) beads [36,37], facilitating the achievement of higher local antibiotic concentrations. Absorbable mineral-based materials are not as mechanically strong as acrylic cements, but they provide some advantages for antibiotic release and infection control. They release the full antibiotic load and achieve high levels of antibiotics locally over sustained periods, leaving no residual foreign body that could act as a nidus for biofilm formation. We have previously studied the killing of biofilms of bioluminescent strains of *P. aeruginosa* (PA-Xen41) and *S. aureus* (SA-SAP231) grown on agar surfaces by a combination of vancomycin and tobramycin impregnated calcium sulfate beads for three days [38]. In this present study, we extended the incubation period to determine whether we could achieve complete eradication of biofilm bacteria adjacent to the beads, including resistant phenotypes that develop in the presence of antibiotics. We used the replica plating technique onto non-antibiotic agar to allow persister cells, if present, to grow. In addition, we assessed whether the spatial pattern of the beads was important to completely eradicate the biofilms grown on agar surfaces by systematic arrangement of antibiotic-loaded calcium sulfate beads (ALCSBs) since clinically beads sprinkled into the surgical site show different densities and patterns of clustering [36,39]. The overall goal of this study was to determine whether the arrangement of antibiotic loaded beads was an important factor in completely eradicating biofilms of PA and SA in a simple in vitro assay.

## 2. Methods

### 2.1. Bacterial Strain and Culture Conditions

Bioluminescent strains of *P. aeruginosa* PA-Xen41 [40] (Perkin-Elmer, Waltham, MA, USA) and USA300 *S. aureus* SA-SAP231 [40] were used. The glycerol stock cultures were stored at −80 °C and streaked onto fresh tryptic soy agar (TSA) and brain heart infusion (BHI) agar for PA and SA, respectively, and incubated for 24 h. The isolated colonies from the respective plates were transferred aseptically to 20 mL of TSA and BHI broth using a sterile inoculating loop and incubated overnight on a shaker incubator set at a temperature of 37 °C and a speed of 200 rpm. These bioluminescent strains have been genetically engineered to constitutively give off light when they are metabolically active and as such are useful tools to non-invasively track temporal and spatial changes in biofilm activity.

### 2.2. Preparing Lawn Biofilms

Lawn biofilms of PA-Xen41 and SA-SAP231 were prepared by spreading the overnight cultures onto TSA and BHI agar respectively, unless otherwise stated. Briefly, 100 µL of the overnight culture was mixed with 9.9 mL of liquid medium to make a 1:100 dilution. 200 µL of the diluted culture was spread onto 90 mm diameter polystyrene Petri dishes (Thermo-Fisher Scientific, Waltham, MA, USA) containing TSA or BHI agar. The plates were incubated at 37 °C for 24 h to develop lawn biofilms [41].

### 2.3. Preparation of Antibiotic-Loaded Calcium Sulfate Beads (ALCSB)

ALCSBs were prepared using 10 cc Stimulan^®^ Rapid Cure as per directions from Biocomposites Ltd. (Keele, Staffordshire, UK) and in previously described methods [38]. Tobramycin (240 mg, Sigma-Aldrich, St. Louis, MO, USA) and vancomycin (1000 mg, Sigma-Aldrich, St. Louis, MO, USA) combinations were used to prepare the beads [36,38]. ALCSBs of 4.8 mm diameter were prepared using standard mold mats (Biocomposites Ltd, Keele, UK). The beads were left to cure for 1 h and removed from the mold mats by manual twisting. ALCSB were stored at 4 °C until use in the experiments. Vancomycin and tobramycin are commonly incorporated into bone cement and Stimulan absorbable bone filler to provide broad coverage of Gram-positive and -negative pathogens commonly associated with PJI [37,42].

### 2.4. Killing of Lawn Biofilms Using Antibiotic Beads

A combination of vancomycin and tobramycin was used to examine the killing of biofilms of PA-Xen41 and SA-SAP231. Briefly, after generation of a 24 h lawn biofilm on agar using the method described above, the ALCSB (containing vancomycin and tobramycin) were placed in the center of the plates containing pre-grown 24 h lawn biofilms of PA-Xen41 or SA-SAP231 using sterile forceps. ALCSBs were gently pushed into the agar to allow uniform diffusion of antibiotics throughout the medium. Plates were incubated at 37 °C with 5% CO_2_ for seven days and images (both normal digital photographs and in vivo imaging system [IVIS]) were captured every day. The IVIS system has a very sensitive photon detector that can capture the low levels of light produced by the bioluminescent strains of bacteria. The relative brightness in an image is captured as a grey scale ranging from white (very bright indicating relatively high metabolic activity) to black indicating no detection of metabolic activity. To enhance visual detection of features, the greyscale image is false colored to convert to a “heat map” in which red is mapped to white showing the highest level of metabolic activity with blue being close to black indicating low relative metabolic activity and black indicating no detected light. Since lack of metabolic activity does not necessarily mean that the cells in the biofilm are dead, a culturing technique (replica plating in the present study) is required to assess viability.

### 2.5. Killing of Lawn Biofilms by Antibiotics Impregnated on Filter Paper Discs

To determine whether the antibiotic carrier material had a role in the growth of the antibiotic resistant phenotypes seen with PA-Xen41, filter paper discs (6 mm, Sigma Aldrich, St. Louis, MO, USA) were impregnated with tobramycin and used instead of ALCSB. Tobramycin alone at a concentration of 100 µg/disc was used. Since PA-Xen41 was not susceptible to vancomycin, thus only tobramycin was used. The discs were placed on the pre-grown lawns of PA-Xen41 and incubated for seven days, and IVIS images were captured every day. For comparative purposes we also used ALCSB loaded with tobramycin alone (240 mg/10 cc pack).

### 2.6. Effect of ALCSB Arrangements on Killing of Lawn Biofilms

ALCSB containing vancomycin and tobramycin were placed on 24 h biofilms of PA-Xen41 and SA-SAP231 as: (i) a single bead in the center, sixteen beads placed as (ii) sets of four beads (iii) circularly, close to the edges of the plates, (iv) in the center, and (v) 19 beads placed hexagonally equidistant. For the hexagonal bead placement patterns, we used 19 beads, since previously we found that the area of the zone of biofilm killing (ZOB-K) for a single ALCSB was 1.8 cm^2^, with the intention that zones of biofilm killing would overlap. These patterns were chosen to represent a range of different possibilities of clustering patterns that might occur from the manual sprinkling of beads into the surgical site; in this situation, unless there are enough beads to completely fill the site, inevitably, there will be some clustering while some beads remain more isolated [36,39]. The ZOB-K and the appearance of antibiotic-resistant phenotypes were monitored daily using IVIS and white light camera imaging.

### 2.7. Determination of Killing of Biofilms Using Replica Plating Technique

Spatial killing of lawn biofilms of PA-Xen41 and SA-SAP231 was determined using a traditional replica plating technique with minor modifications [43]. Briefly, a sterile cotton velveteen square cloth (150 mm by 150 mm) was aseptically draped over a PVC Science-ware replica plater (Sigma-Aldrich, St. Louis, MO, USA) and locked in place with an aluminum ring. ALCSBs were removed from the plate at day seven using sterile forceps. Plates were inverted over the velveteen cloth and tapped gently to ensure complete contact of the lawn biofilm with the cloth. A sterile plate containing TSA with tobramycin (5 µg/mL) was also inverted onto the velveteen cloth containing the previously stamped cells. Secondly, a fresh TSA containing plate without antibiotics was placed on the velveteen cloth and tapped gently to ensure complete surface contact. Both replica plated plates were incubated for five days at 37 °C in an incubator with 5% CO_2_ to allow for the appearance of slow growing colonies. Colonies that grew out from the lawn and on the replica-plates were presumed to be resistant phenotypes. Colonies that appeared on the replica plates (without antibiotic) but not on the original tobramycin-containing plates were presumed to be persister cells [44,45,46,47,48].

### 2.8. Image Analysis to Determine the Influence of Bead Number on Killing of Lawn Biofilms

IVIS images of lawn biofilms of PA-Xen41 and SA-SAP231 subjected to ALCSB containing vancomycin and tobramycin at 24 h were subjected to image analysis using ImageJ (Version 1.51h) [49]. The plot profile function was used to measure the distance between the cleared edge and the peripheral bead. A circular region of interest (ROI) was used to measure the cleared area and the area of a single bead (20.1 ± 2.3 mm^2^, n = 4, equivalent to a diameter of 4.94 mm, very close to mold size of 4.8 mm). Distance cleared from the bead, area cleared, and area cleared per bead of single and clusters of four and 16 ALCSB were compared.

### 2.9. Antibiotic Carryover During Replica Plating

To determine whether there was a carryover of antibiotic by velveteen cloth during replica plating, the ALCSBs were placed on a TSA plate for five days and replica plated onto fresh TSA plates. The replica plates were then spread with PA-Xen41 and were incubated at 37 °C in an incubator with 5% CO_2_.

### 2.10. Estimation of Antibiotic Concentration Eluted

Tobramycin-loaded beads were placed in the sterile agar plates. Plates were incubated at 37 °C with 5% CO_2_ and at various time points, plates were removed and marked with 5 mm by 5 mm squares from the edge of the bead to the edge of the plate. Each of these squares was excised using a sterile razor blade and forceps. The excised plugs were melted at 80 °C in a water bath. An overnight culture of *P. aeruginosa* PAO1 was diluted to an optical density measured at a wavelength of 600 nm OD_600_) of 0.1 and spread onto sterile TSA plates. A sterile filter paper disc was then placed in the center of the plate and 10 µL of the melted plug was placed onto the paper disc. The plates were incubated for 24 h at 37 °C with 5% CO_2_. After incubation, the zones of inhibition were measured and compared to a standard curve for tobramycin prepared in TSA to determine the concentration of antibiotic in the melted plug. This process was repeated at the various time points, using different plates each time.

## 3. Statistical Analysis

All experiments were performed in triplicate. Control and treated samples were compared by *t*-test, assuming equal variance. Student’s *t*-test was used for all other comparison of differences between means, whereby *p* < 0.05 was considered significant. Data represented are plotted as mean ± SD.

## 4. Results

### 4.1. Killing of Lawn Biofilms by ALCSB

**Figure 1:**
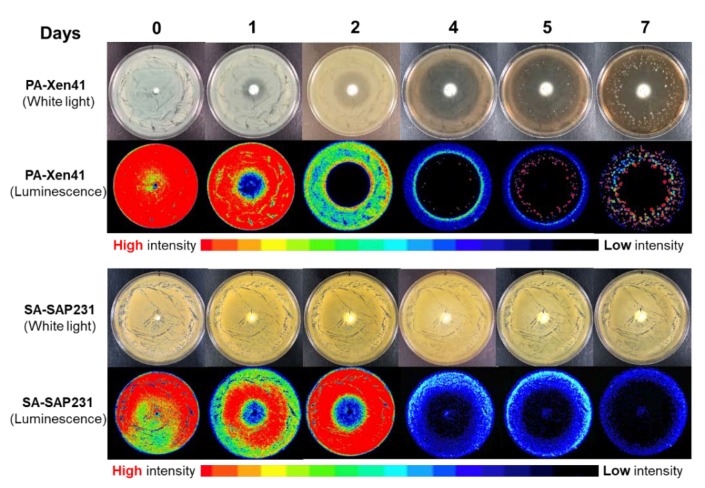
Killing of lawn biofilms of *P. aeruginosa* (PA-Xen41) and *S. aureus* (SAP231) with vancomycin and tobramycin loaded beads at the center. Zone of biofilm killing (ZOB-K) was monitored every day for seven days, and antibiotic-resistant phenotypes were observed in PA-Xen41 beginning at day four.

Bioluminescent strains of PA-Xen41 and SA-SAP231 were used in this study. The use of bioluminescent strains has the advantage of enabling identification of the zone of biofilm killing and the growth of antibiotic-resistant phenotypes on the lawn biofilms, which are otherwise difficult to differentiate with normal photographs (Figure 1). The previously established method for lawn biofilm formation was used, which considers an immobile community of bacteria attached to agar surface, embedded in an exo-polymeric substance (EPS), with high cell number and exhibiting tolerance towards antibiotics [41]. The initial cell concentrations of the 24 h lawn biofilms were in the range of 1 ± 3 × 10^9^ CFU/mL. The minimum inhibitory concentration (MIC) of both vancomycin and tobramycin against SA-SAP231 was 2.0 μg/mL and for PA-Xen41, the MIC of tobramycin was 1.5 μg/mL. Previous reporting has shown that vancomycin had no effect on the growth of PA-Xen41 [38]. Clinically used combinations of antibiotics (vancomycin and tobramycin) eluting from the ALCSB showed an increase in the zone of killing of biofilms over time (Figure 1). The zone of killing was observed in the case of both PA-Xen41 and SA-SAP231 biofilms, with rapid killing of PA-Xen41 as compared to SA-SAP231 biofilms (Figure 1). PA-Xen41 is not sensitive to vancomycin; therefore, the zone of biofilm killing with vancomycin was not determined against PA-Xen41.

### 4.2. Evidence of Antibiotic-Resistant Phenotypes

**Figure 2:**
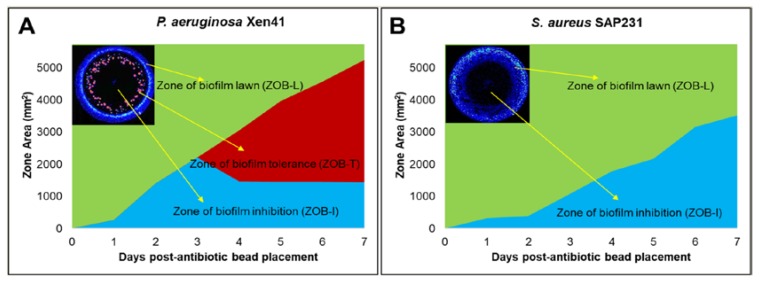
Different zones were developed after placement of vancomycin and tobramycin loaded beads on lawn biofilms of (**A**) PA-Xen41 and (**B**) SA-SAP231. Zone of biofilm killing (ZOB-K), zone of biofilm resistance (ZOB-R), and zone of biofilm lawn (ZOB-L) were evident over time after antibiotic bead placement. Images in insets show vancomycin and tobramycin treated day five lawn biofilms of Xen41 (**A**) and day six lawn biofilms of SAP231 (**B**).

Antibiotic-resistant phenotypes were observed with vancomycin and tobramycin against PA-Xen41 (Figure 1 and Figure 2A). These phenotypes were not evident in SA-SAP231 when treated with vancomycin and tobramycin (Figure 1 and Figure 2B). Three different zones were evident on biofilms of PA-Xen41 treated with ALCSB; (i) the zone of the edge of the still active biofilm lawn (ZOB-L), (ii) zone of biofilm resistance (ZOB-R), and (iii) the zone of biofilm killing (ZOB-K), (Figure 2A). SA-SAP231 biofilms treated with vancomycin and tobramycin had two zones (Figure 2B), the ZOB-L, ZOB-K but no ZOB-R. Antibiotics, vancomycin and tobramycin containing ALCSB showed the killing of PA-Xen41 for three days. Antibiotic resistant colonies emerged beginning at day four, that were clearly visible thereafter (Figure 1). The generation of antibiotic resistant colonies was evident away from the ALCSBs with the zone of killing towards the area surrounding the ALCSB. The ZOB-R followed the ZOB-K which showed the presence of antibiotic resistant colonies and the ZOB-L that diminished over time (Figure 2A). ZOB-R increased over time suggesting the rapid growth of antibiotic-resistant cells within the area of ZOB-K, where antibiotics gets depleted. Tobramycin alone showed similar tolerant zones on PA-Xen41 lawn biofilms at day four as observed with vancomycin and tobramycin (Figure S1). In the case of SA-SAP231, vancomycin and tobramycin had no tolerant phenotypes; however, with tobramycin alone antibiotic-resistant colonies were evident at day six (data not shown). Using the filter paper discs as antibiotic carrier, antibiotic-resistant phenotypes were also evident as with ALCSB. The generation of antibiotic resistant phenotypes were independent of the carrier material, whether tobramycin-impregnated filter paper discs or ALCSBs (Figure S1). From the IVIS images, we estimated that the concentration of these residual viable phenotypes in the PA zone of lawn clearing was approximately 10 CFU/cm^2^. Since the initial 24 h lawn contained approximately 1.2 × 10^6^ CFU/cm^2^ [40], these represent a very low frequency of approximately 1:100,000 and may easily be missed by conventional clinical methods or a conventional Kirby-Bauer assay, which has a much lower initial concentration of cells on a plate (i.e., 100 µL of approximately 10^5^ CFU/mL spread on a normal 100 mm diameter plate would be approximately 1000 CFU/cm^2^).

### 4.3. Killing of PA-Xen41 and SA-SAP231 Lawn Biofilms by Systematic Arrangement of ALCSB

**Figure 3:**
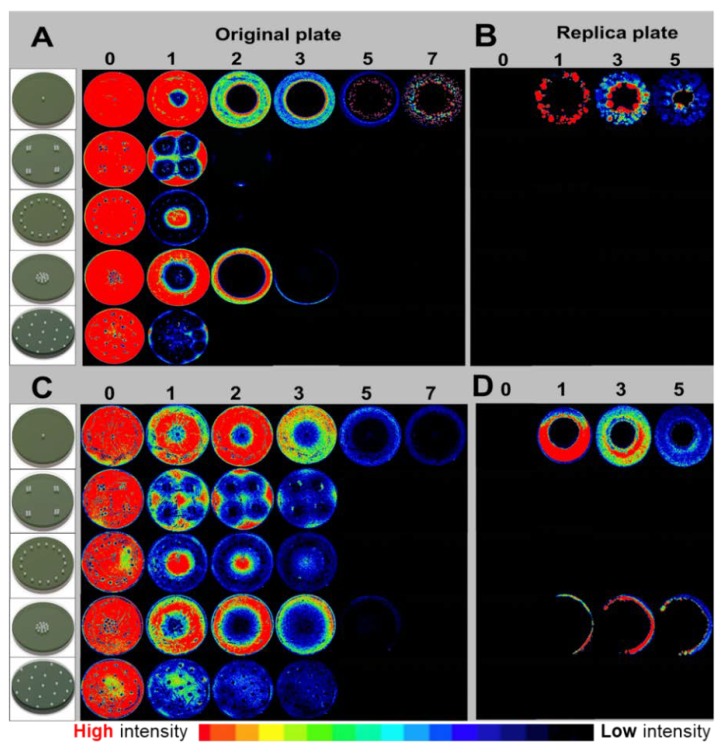
Effect of bead placement on killing of PA-Xen41 (**A**,**B**) and SA-SAP231 (**C**,**D**) biofilms treated with vancomycin and tobramycin, where (**A**,**C**) are original plates and (**B**,**D**) are replica plates of original petri-plates at day seven.

**Figure 4:**
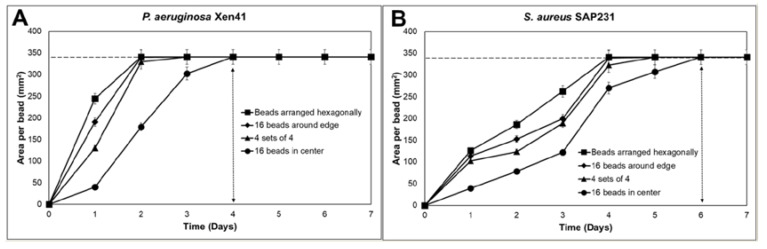
Killing of lawn biofilms of (**A**) PA-Xen41 and (**B**) SA-SAP231 treated with vancomycin and tobramycin. Dotted horizontal lines in the graphs represents edges of the Petri dishes and vertical lines represents days of complete lawn biofilm killing.

The ALCSBs containing vancomycin and tobramycin were arranged on the lawn biofilms of PA-Xen41 and SA-SAP231 as: (i) a single bead in the center, (ii) 16 beads placed as four clusters of four, (iii) 16 beads placed in a ring, (iv) 16 beads placed as a group in the center, or (v) 19 beads placed evenly across the Petri dishes (Figure 3). Compared to a single bead with vancomycin and tobramycin, all four bead arrangements were more effective in killing biofilms of PA-Xen41 (Figure 3A,B) and SA-SAP231 (Figure 3C,D). The PA-Xen41 lawn biofilms were rapidly killed at day three, except for the 16 beads when placed in the center, which showed loss of activity by IVIS at day four (Figure 4A). With all the bead placements, the killing of biofilms of PA-Xen41 at days one and two and SA-SAP231 at days two, three, and four were significantly different than the beads in the center (*p* < 0.05, Figure 4A,B). In the case of SA-SAP231, the lawns were killed at day five with all the ALCSB arrangements (Figure 4B), except the lawn with 16 beads placed in the center, which showed loss of activity under IVIS at day six. The arrangements of beads closer to the edges, all beads in the center, and the beads placed in hexagonal patterns rapidly eradicated the lawn biofilms within five days for both strains (Figure 4A,B). There could be a possibility of an edge effect, since the beads are very close to the edges of the petri plates, thereby limiting the diffusion of antibiotics. When all the beads were placed in the center, they did not effectively eradicate biofilms in the case of SA-SAP231 (Figure 3D and Figure 4B). The bead placement showed significant killing of biofilms as compared to a single bead. The pattern of ALCSB placement was important in complete killing of lawn biofilms with no generation of antibiotic-resistant colonies (Figure 3A–D).

### 4.4. Replica Plating to Determine Complete Killing or Regrowth after ALCSB Treatment

Replica plating was used to determine whether all the biofilm cells were killed after treatment with ALCSB or if antibiotic-resistant cells would regrow when transferred to fresh plates. When PA-Xen41 and SA-SAP231 treated with vancomycin and tobramycin for seven days were replica-plated onto fresh TSA or BHI agar, growth of antibiotic-tolerant phenotypes was observed with single bead placement in the center (Figure 3B,D). No growth was observed in the area closest to the ALCSB suggesting that this area was completely sterile. All the bacterial cells present in the lawn biofilms close to the ALCSB were killed. For all the bead arrangements, replica plates incubated for five days showed complete killing and sterility due to the high concentration of antibiotics achieved (Figure 3B,D). No carryover of antibiotics was observed during replica plating from ALCSB-containing plates onto fresh agar plates (Figure S2). Lawn biofilms of SA-SAP231 treated with vancomycin and tobramycin for seven days, when replica plated onto BHI agar revealed incomplete killing with a single bead and when 16 beads were placed in the center (Figure 3D). All other bead arrangements were effective in complete killing of the biofilms from the BHI agar containing plates (Figure 3D), as observed with the replica plating technique. This suggests that systematic arrangement of ALCSB, i.e., minimizing the bead separation and maximizing the coverage, is necessary to completely eradicate lawn biofilms as well as prevent growth of antibiotic-resistant cells.

### 4.5. Image Analysis to Determine the Influence of Bead Number on Killing of Lawn Biofilms

**Figure 5:**
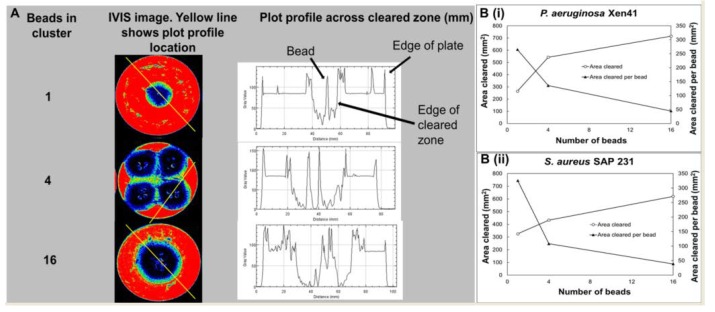
(**A**) Influence of bead number on clearing of lawn biofilms of PA-Xen41 after 24 h of treatment with antibiotic-loaded calcium sulfate beads (ALCSB) containing vancomycin and tobramycin. Image analysis (Image J) of beads in clusters showing profile plot across cleared zone (mm); (**B**) Influence of bead number on total area and area cleared per bead of lawn biofilms of PA-Xen41 (i) and SA-SAP231 (ii) after 24 h of treatment with beads containing vancomycin and tobramycin. Distance cleared from edge of bead cluster was relatively independent of the number of beads. More beads cleared a greater area; however, the area cleared per bead was dramatically reduced when more beads were clustered together.

The influence of bead number on clearing of biofilms was determined using IVIS images of PA-Xen41 and SA-SAP231 treated with ALCSBs containing vancomycin and tobramycin at 24 h (Figure 5A,B). The plot profile across the single, four, or 16 beads clustered together suggested that the distance cleared from the edges of the bead clusters is relatively independent of the number of beads. More beads cleared a greater area, but the area cleared per bead was significantly reduced when more beads were clustered together (Figure 5B). Taken together, beads should be distributed densely and as evenly as possible over the whole biofilm (or at surgical sites) to make sure there are overlapping zones of biofilm killing.

## 5. Discussion

*S. aureus* and *P. aeruginosa* have been implicated in serious infections due to the formation of biofilms that are associated with resistance towards antibiotics [10,11]. Bacterial biofilms are a major concern in patients with PJI, and beads incorporated with antibiotics are routinely used at the site of infection during orthopedic surgeries to provide sustained release and local treatment to prevent infections [34,35,36,37]. While this therapy may be effective in killing a large proportion of bacteria, it may be ineffective against bacteria that are protected within biofilms, or at places where antibiotic concentrations could be low, and at areas away from the beads, potentially resulting in repeated and chronic infections.

We have previously demonstrated the short-term killing of lawn biofilms of PA and SA using ALCSB with combinations of vancomycin and tobramycin for up to three days [38]. In this present study, we were interested in understanding the long-term effect of ALCSB on killing of lawn biofilms; therefore, we extended the exposure time of killing of biofilms by ALCSBs to achieve complete eradication of biofilms grown on agar surfaces. Using the lawn biofilm killing method by ALCSB [38], we observed that PA-Xen41 biofilms were rapidly cleared with vancomycin and tobramycin compared to biofilms of SA-SAP231. Interestingly, in the presence of vancomycin and tobramycin, antibiotic-resistant phenotypes were evident in PA-Xen41 but not in SA-SAP231. To investigate which antibiotic from the combination is responsible for eliciting the generation of these antibiotic-resistant phenotypes, vancomycin and tobramycin were used independently against PA-Xen41. We found that antibiotic-resistant phenotypes were observed growing out of the PA-Xen41 biofilm lawn killed after exposure to tobramycin where the concentrations are much higher than the MIC (Figure S3). Notably, these colonies did not reveal themselves immediately but only after three days when the cells in the background gets cleared; thus, this small population could go undetected without extended incubation.

Tobramycin belongs to the aminoglycoside group of antibiotics. Antibiotic resistance towards aminoglycosides has been observed previously in PA and SA [15,17,18,47]. Different mechanisms have been proposed, as mentioned earlier, for the development of resistance towards antibiotics such as the role of efflux pumps, cell wall permeability, development of robust biofilms, and SCVs. In a recent study, Hoiby et al. (2019) showed that when planktonic bacteria are seeded, they start to grow and form aggregates. After 5 h these aggregates become tolerant to tobramycin and continue to grow and form a mature biofilm after 7 h of incubation, which are completely resistant to tobramycin [33]. These mechanisms contribute to the survival of bacteria to antibiotic treatment and might lead to the treatment failures. Therefore, novel strategies to eradicate biofilms and prevent the development of antibiotic resistance are needed. To achieve complete killing of biofilm lawns and to prevent the resistant phenotypes, we strategically arranged ALCSB containing vancomycin and tobramycin in four different patterns. On the agar lawn biofilms of PA and SA, we compared a single bead against the arrangements with sixteen beads (i) in groups of four, (ii) close to the edge of the plates, (iii) all the beads in the center, and (iv) 19 beads distributed hexagonally. All the arrangement of beads was found effective in killing of biofilms of PA (Figure 3A,B and Figure 4A) and SA (Figure 3C,D and Figure 4B). However, 16 beads clustered together in the center were not effective in completely eradicating the lawn biofilms of SA (Figure 3D). Importantly, in PA no colonies grew out of the zone of biofilm killing (Figure 3A,B). Replica plating suggested that all the cells were killed (Figure 3A–D).

These antibiotic bead placement strategies could be effective in cases where infections are not cleared efficiently because of difficulty in accessing the site of infection, such as in the case of device-associated joint infections. Furthermore, replica plating of these biofilms treated with ALCSB demonstrated complete killing of lawns and revealed that the inner concentration of antibiotics was high enough to completely kill the biofilms present in the areas close to the ALCSB (Figure 3A–D and Figure 4A,B).

It is important to note that high concentration of antibiotics is also able to prevent the growth of resistant phenotypes. This is indicated by the lack of growth in the zone of biofilm killing on replica plates as well as by the lack of growth in cultures taken from the zones immediately surrounding the beads, where high concentration of antibiotics was present (Figure 2A,B and Figure S3). Total eradication of biofilms in orthopedic infections by systemic administration of antibiotics is extremely challenging, since the concentrations required to eradicate biofilms of clinical strains are often greater than 1000 µg/mL for vancomycin as well as a wide range of antibiotic classes [14,48], in part due to the presence of persister cells [49]. However, concentrations of vancomycin achieved in the serum and knee joint fluid by IV administration are much lower, approximately 25 and 7 µg/mL, respectively [50]. Castaneda et al. [51] has shown that biofilms can be eradicated, presumably including the killing of persister populations, using high concentrations over extended incubation periods [51]. Our data are in agreement, however; we did find differences in the rate of clearing, with the 16 beads clustered in the center taking longer to clear the biofilms than those that were distributed around the Petri dishes. The hexagonally placed beads showed the most rapid killing compared to other combinations. Similar results were seen with SA, although the beads clustered in the center did not killed all the biofilms at the periphery of the plate as is evidenced by growth on the replica plates (Figure 3D). Our data suggest that in diffusion-dominated areas, it is not only the number of beads placed at the site that is important for controlling biofilm, but also that the beads have enough density and are distributed such that the concentration and duration of antibiotic exposure are sufficient to completely kill the biofilm cells in the vicinity of the beads. Our observations do not only apply to beads but to any carrier that is releasing antibiotics, since spatial and temporal concentration gradients will always develop, although the scales of these gradients will strongly depend on the carrier and local mass transfer conditions. The proximity of the beads to each other and to the infected material should be based not just on the area of the zone of biofilm killing, which is seen in the first 24 h, but also at later times in this zone in which bacterial resistant colonies only reveal themselves after extended incubations. Therefore, closely packed ALCSBs with enough coverage of areas suspected of infection might be effective in completely eradicating PA and SA biofilms at the surgical site, overcoming the problem of generation of antibiotic-resistant bacteria that could otherwise escape antibiotic treatment and regrow to establish biofilm-related infections.

In future studies, we will focus on the specific mechanisms underlying the generation of antibiotic-resistant phenotypes and determine whether these phenotypes are observed within a particular class of antibiotics and whether different combination of antibiotics could prevent the development of these antibiotic-resistant phenotypes that could potentially regrow and cause infection in the clinical settings.

## 6. Conclusions

We have shown that: (1) the antibiotics released from ALCSB can totally eradicate in vitro biofilms, including resistant population growing within the zones of biofilm killing around each bead; (2) in PA, antibiotic resistant phenotypes may survive outside the zone of biofilm killing; and (3) the distribution and the number of beads are important to ensure adequate coverage in order to eradicate in vitro biofilms from a given area.

## Supplementary Materials

The following are available online at https://www.mdpi.com/1996-1944/12/24/4052/s1, Figure S1: Killing of lawn biofilms of *P. aeruginosa* (PA-Xen41) with antibiotic-loaded calcium sulfate beads (ALCSB) and paper discs loaded with tobramycin. The ALCSB loaded with 240 mg/10 cc tobramycin and filter paper discs with 100 µg/disc tobramycin were placed in the center on 24 h lawn biofilms. Zone of biofilm killing (ZOB-K) and resistant colonies were observed with both ALCSB and paper discs loaded with tobramycin, Figure S2: Carryover of antibiotic during replica plating. ALCSB containing tobramycin placed on 24 h grown lawn biofilms at day five was replica plated onto fresh tryptic soy agar (TSA) plates and spread with PA-Xen41. No inhibition of growth of PA-Xen41 was observed, suggesting no carryover of tobramycin during replica plating, Figure S3: Tobramycin concentration remains above MIC during resistant phenotype development. Tobramycin-loaded bead was placed in sterile TSA, and at various time points, agar plugs were extracted at various radii to examine the concentration of tobramycin in them by plating for MIC (n = 3). MIC zones were compared to a standard curve to calculate the tobramycin concentrations in the agar plugs. Data is reported as mean ± SD.
