# Supplementary material for: Complete Killing of Agar Lawn Biofilms by Systematic Spacing of Antibiotic-Loaded Calcium Sulfate Beads
Source: Materials (Basel). 2019 Dec 5;12(24):4052. doi: 10.3390/ma12244052 (PMC6947297; doi:10.3390/ma12244052)
Supplement: Supplementary file 1 [file materials-12-04052-s001.pdf]

materials

Supplementary Materials

Complete Killing of Agar Lawn Biofilms by Systematic Spacing of Antibiotic-Loaded Calcium Sulfate Beads

Devendra H. Dusane [1], Jacob R. Brooks [1], Devin Sindeldecker [1], Casey W. Peters [1], Anthony Li [1], Nicholas R. Farrar [1], Scott M. Diamond [1], Cory S. Knecht [1], Roger D. Plaut [2], Craig Delury [3], Sean S. Aiken [3], Phillip A. Laycock [3], Anne Sullivan [4], Jeffrey F. Granger [4] and Paul Stoodley [1,4,5,*,†]

[1] Department of Microbial Infection and Immunity, The Ohio State University, Wexner Medical Center, Columbus, OH 43210, USA 43210; devendra.dusane@osumc.edu (D.D.); brooks.922@buckeyemail.osu.edu (J.B.); sindeldecker.3@osu.edu (D.S.); peters.690@osu.edu (C.P.); li.5960@osu.edu (A.L.); Nicholas.Farrar@osumc.edu (N.F.); Scott.Diamond@beaumont.org (S.D.); knechtc2@ccf.org (C.K.)

[2] Division of Bacterial, Parasitic, and Allergenic Products, Center for Biologics Evaluation and Research, Food and Drug Administration, Silver Spring, MD, 20993, USA; Roger.Plaut@fda.hhs.gov

[3] Biocomposites Ltd., Keele Science Park, Keele, Staffordshire ST5 5NL, UK; cpd@biocomposites.com (C.D.); sa@biocomposites.com (S.A.); pl@biocomposites.com (P.L.)

[4] Department of Orthopaedics, The Ohio State University, Wexner Medical Center, Columbus, OH 43210, USA; anne.sullivan@osumc.edu (A.S.); jgranger230@gmail.com (J.G.)

[5] National Centre for Advanced Tribology at Southampton (nCATS) and National Biofilm Innovation Centre (NBIC), Department of Mechanical Engineering, University of Southampton, Southampton, SO17 1BJ, UK

* Correspondence: paul.stoodley@osumc.edu.com; Tel.: +1614-292-7871

† Department of Microbial Infection and Immunity, The Ohio State University, 760 Biomedical Research Tower, 460 West, 12th Avenue, Columbus 43210, OH, USA

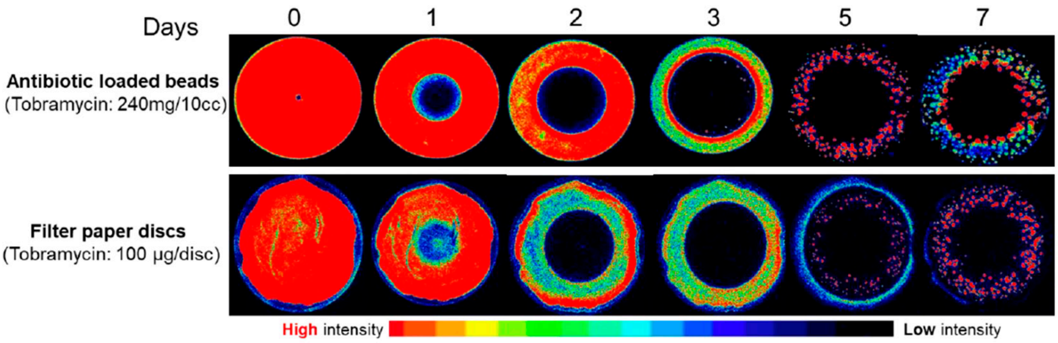

Figure S1. Killing of lawn biofilms of P. aeruginosa (PA-Xen41) with antibiotic-loaded calcium sulfate beads (ALCSB) and paper discs loaded with tobramycin. The ALCSB loaded with 240 mg/10 cc tobramycin and filter paper discs with 100 µg/disc tobramycin were placed in the center on 24 h lawn biofilms. Zone of biofilm killing (ZOB-K) and resistant colonies were observed with both ALCSB and paper discs loaded with tobramycin.

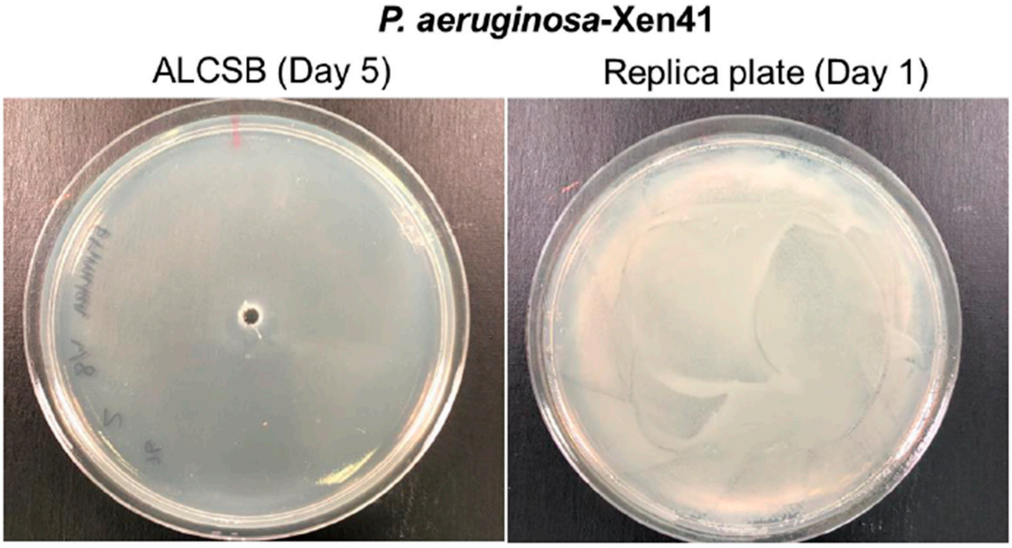

Figure S2. Carryover of antibiotic during replica plating. ALCSB containing tobramycin placed on 24 h grown lawn biofilms at day five was replica plated onto fresh tryptic soy agar (TSA) plates and spread with PA-Xen41. No inhibition of growth of PA-Xen41 was observed, suggesting no carryover of tobramycin during replica plating.

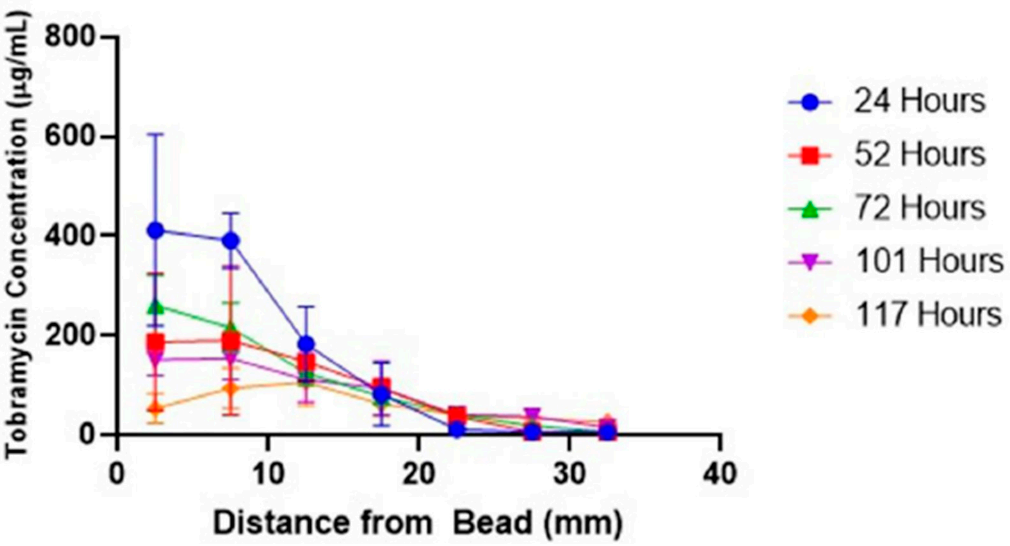

Figure S3. Tobramycin concentration remains above MIC during resistant phenotype development. Tobramycin-loaded bead was placed in sterile TSA, and at various time points, agar plugs were extracted at various radii to examine the concentration of tobramycin in them by plating for MIC (n = 3). MIC zones were compared to a standard curve to calculate the tobramycin concentrations in the agar plugs. Data is reported as mean ± SD.
